# Supplementary material for: Three-Dimensional Model of Dorsal Root Ganglion Explant as a Method of Studying Neurotrophic Factors in Regenerative Medicine
Source: Biomedicines. 2020 Mar 3;8(3):49. doi: 10.3390/biomedicines8030049 (PMC7175199; doi:10.3390/biomedicines8030049)
Supplement: Supplementary file 1 [file biomedicines-08-00049-s001.zip › Supplement.docx]

**TROUBLESHOOTING**

Troubleshooting advice can be found in Table S5 below:

TABLE S5

| **Step** | **Problem** | **Possible reason** | **Solution** |
| --- | --- | --- | --- |
| 1 | No neurites outgrowing from the DRG can be seen. | Neurites are exclusively formed by neurons that are located in the ganglia. If no neurites can be seen in the Matrigel on the 4^th^ day, most likely there was an error at the stage of tissue isolation (e.g., part of the spinal cord or adipose tissue was isolated instead of DRG). | Run the protocol again. |
| 2 | Matrigel with DRG explant is not transparent. | DRG isolating was not performed in sterile conditions, contamination occurred. | Run the protocol again, paying a special attention to sterile conditions. |
| 3 | A drop of Matrigel detached from the well. | When changing the medium, a new portion was applied too quickly or the jet was too strong. Otherwise the aspirator touched the Matrigel. | Transfer the pop-up Matrigel with the DRG to a new well. Before the transfer, drop 10μl of Matrigel onto the bottom of the new well. Carefully put the plate into the CO_2_ incubator to allow Matrigel polymerization. Then add the required volume of culture medium. |
| 4 | Bubbles in Matrigel. | When defrosting, Matrigel was shaken. | Place Matrigel on ice. Wait until air bubbles pop up. Take the Matrigel from the bottom of the ampoule. |
