# Supplementary figures and images for: Three-Dimensional Model of Dorsal Root Ganglion Explant as a Method of Studying Neurotrophic Factors in Regenerative Medicine
Source: Biomedicines. 2020 Mar 3;8(3):49. doi: 10.3390/biomedicines8030049 (PMC7175199; doi:10.3390/biomedicines8030049)

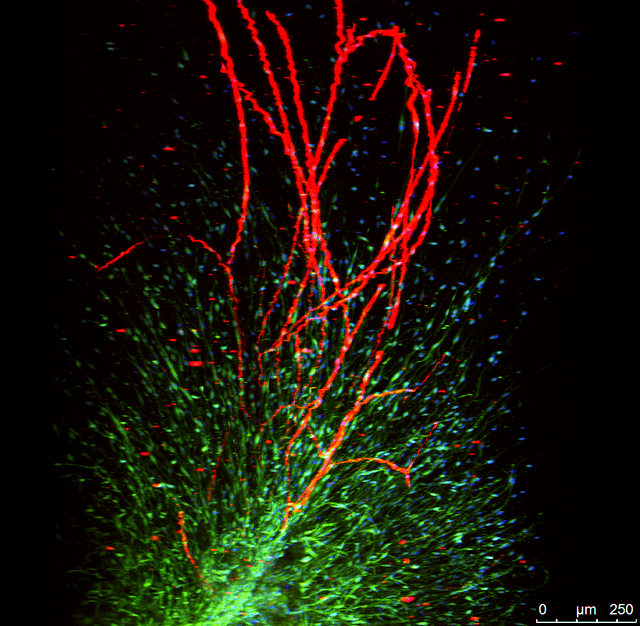

Supplement: Supplementary file 1 [file biomedicines-08-00049-s001.zip › Video S1.gif]

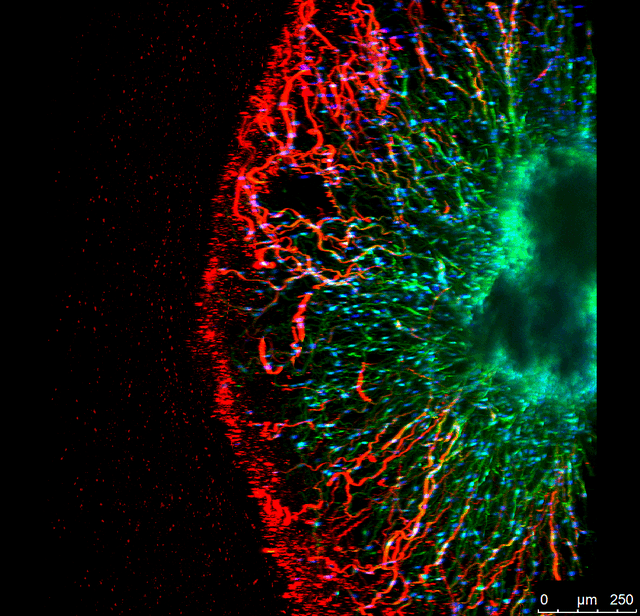

Supplement: Supplementary file 1 [file biomedicines-08-00049-s001.zip › Video S2.gif]

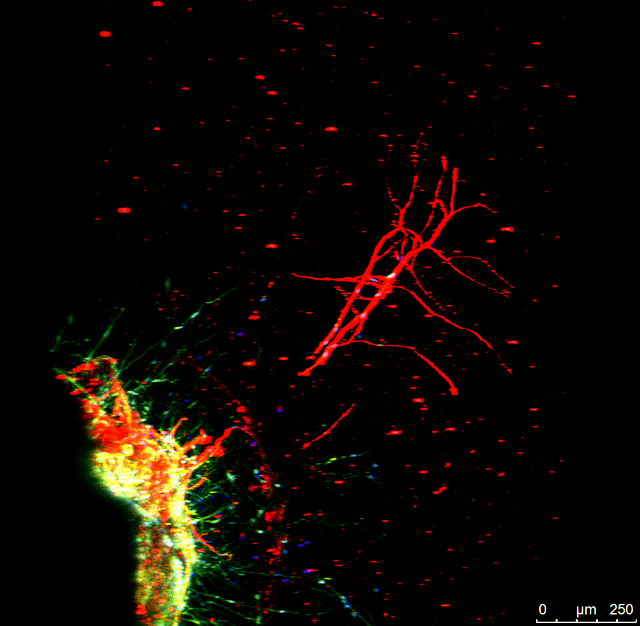

Supplement: Supplementary file 1 [file biomedicines-08-00049-s001.zip › Video S3.gif]

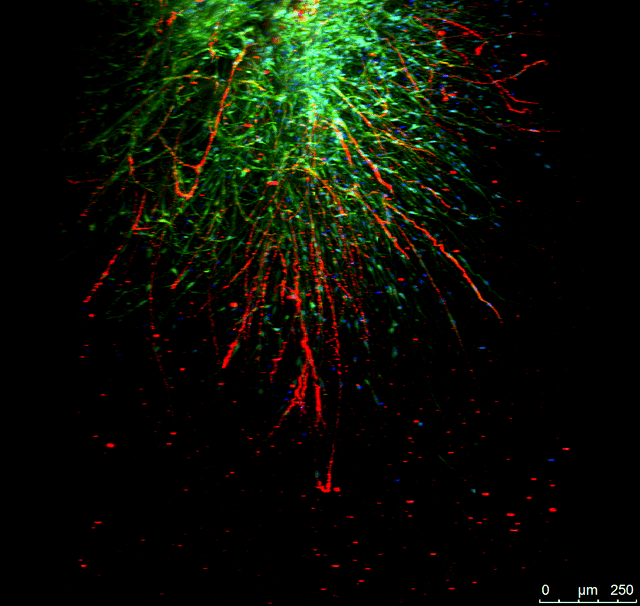

Supplement: Supplementary file 1 [file biomedicines-08-00049-s001.zip › Video S4.gif]
